# Thymidine phosphorylase mRNA expression may be a predictor of response to post-operative adjuvant chemotherapy with S-1 in patients with stage III colorectal cancer

**DOI:** 10.3892/ol.2014.2574

**Published:** 2014-09-29

**Authors:** MASAICHI OGAWA, MICHIAKI WATANABE, YOSHINOBU MITSUYAMA, TADASHI ANAN, MASAHISA OHKUMA, TETSUYA KOBAYASHI, KEN ETO, KATSUHIKO YANAGA

**Affiliations:** Department of Surgery, The Jikei University School of Medicine, Tokyo 105-8461, Japan

**Keywords:** thymidine phosphorylase, S-1, adjuvant chemotherapy, colorectal cancer

## Abstract

The aim of the present study was to investigate markers in surgically resected specimens of colorectal cancer that can be used to predict the response to chemotherapy. The mRNA expression levels of enzymes involved in 5-fluorouracil (5-FU) metabolism and folate metabolism were measured in formalin-fixed, paraffin-embedded tumor sections obtained from the primary tumors of 54 patients with resected stage II or III colorectal cancer who received S-1 for one year. The 5-FU metabolizing enzymes studied were thymidylate synthase, dihydropyrimidine dehydrogenase and thymidine phosphorylase (TP). The folate metabolizing enzymes studied were folypolyglutamate synthetase, γ-glutamyl hydrolase and dihydrofolate reductase. The associations between the mRNA expression levels of these enzymes and clinical variables were investigated. Tumors were classified as exhibiting high or low expression as compared with the median mRNA expression level of each metabolizing enzyme defined as the cutoff value. The associations between the high and low expression levels of each enzyme and disease-free survival (DFS) were analyzed with the use of Kaplan-Meier curves and the log-rank test. DFS was not significantly associated with the relative mRNA expression level of any metabolizing enzyme in the study group as a whole, but there was a trend toward longer DFS in patients with high TP expression (P=0.066). In patients with stage III colorectal cancer, high TP expression was associated with significantly improved outcomes compared with low TP expression (P=0.039). These results indicate that the mRNA expression of TP, a metabolizing enzyme of 5-FU, is a significant predictor of response to post-operative chemotherapy with S-1 in patients with stage III colorectal cancer.

## Introduction

5-Fluorouracil (5-FU) continues to play a central role in chemotherapy for colorectal cancer. S-1 is an oral 5-FU-based anticancer drug that was developed in Japan. This drug combines tegafur, a prodrug of 5-FU, with gimeracil, a reversible antagonist of the rate-limiting enzyme of the metabolic pathway of 5-FU, and oteracil potassium, which is distributed at high concentrations in the gastrointestinal tract, where it reduces gastrointestinal toxicity by irreversibly inhibiting the phosphorylation of 5-FU. The molar ratio of tegafur, gimeracil and oteracil potassium in S-1 is 1.0:0.4:1.0 (1,2). Clinically, the time course of serum 5-FU concentrations following the oral administration of S-1 has been confirmed to be similar to that during a continuous infusion of 5-FU (3). Late phase II studies of patients with advanced or recurrent colorectal cancer have reported a response rate of 37.4% in patients with advanced or recurrent colorectal cancer (4,5). As a second-line chemotherapy for metastatic colorectal cancer, combination therapy with S-1 and irinotecan was shown to be non-inferior to a folinic acid, 5-FU and irinotecan (FOLFIRI) regime (6). Clinical trials have demonstrated that administering S-1 for one year is as effective as post-operative adjuvant chemotherapy in stage II or III gastric cancer. Together with the results of our previous feasibility study, this suggests that S-1 is a promising drug for adjuvant therapy in patients with stage II or III colorectal cancer (7–9).

Recent studies have provided evidence that the expression of enzymes involved in 5-FU metabolism is associated with treatment response (10,11). Thymidylate synthase (TS), a rate-limiting enzyme in DNA synthesis, dihydropyrimidine dehydrogenase (DPD), an enzyme participating in the catabolism of 5-FU, and thymidine phosphorylase (TP), an important metabolizing enzyme of 5-FU, have been studied as predictors of the response or sensitivity to anticancer agents. Enzymes involved in folate metabolism, including folypolyglutamate synthetase (FPGS), γ-glutamyl hydrolase (GGH) and dihydrofolate reductase (DHFR), have been reported to participate in the response to 5-FU-based agents. S-1 is an oral 5-FU-based anticancer drug for which it is considered meaningful to study the value of enzymes involved in 5-FU metabolism or folate metabolism as predictors of treatment response or drug sensitivity.

In the present study, the mRNA expression levels of enzymes involved in 5-FU metabolism or folate metabolism were measured, using tumor specimens obtained from patients with stage II or III colorectal cancer who received post-operative adjuvant chemotherapy with S-1 for one year. The aim of the study was to assess the value of such expression levels as predictors of the response or sensitivity to S-1.

## Materials and methods

### Patients and treatment

The study group was comprised of patients in whom stage II or III colorectal cancer was diagnosed (Union for International Cancer Control staging, sixth edition) (12) and curatively resected in The Jikei University School of Medicine between February 2004 and June 2006. Patients received oral S-1 (Taiho Pharmaceutical Co., Ltd., Tokyo, Japan) as post-operative adjuvant chemotherapy. The daily dose of S-1 (80, 100 or 120 mg per day) was calculated according to body-surface area and administered in two divided doses, one after breakfast and the other after dinner, for 28 consecutive days, followed by a 14-day rest period. The dose of S-1 was 80 mg/day if the body-surface area was <1.25 m^2^, 100 mg/day if the body-surface area was 1.25 to <1.5 m^2^ and 120 mg/day if the body-surface area was ≥1.5 m^2^. The duration of treatment with S-1 was one year. This study was approved by the ethical committee of The Jikei University School of Medicine (Tokyo, Japan) and written informed consent was obtained from all patients.

### Laboratory analysis

#### Expression

Tumor tissue expression levels of various metabolizing enzymes were measured using pathological specimens of resected colorectal cancer. Gene mRNA expression levels of the TS, DPD, TP, FPGS, GGH and DHFR enzymes were semiquantitatively measured by the Dannenberg tumor profiling (DTP) method, and the associations between such levels and clinical variables were studied. An outline of the Dannenberg tumor profiling method is presented below.

#### Staining of formalin-fixed, paraffin-embedded (FFPE) tissue sections

To confirm the site of the tumors, 5-μm thick FFPE tissue sections were stained with hematoxylin and eosin (HE), and the site of cancer was identified and marked by a pathologist. Additionally, 10-μm thick FFPE tissue sections were stained with nuclear fast red (NFR) for RNA extraction.

#### Slicing of cancer tissue

Cancer tissue within the tumor, as designated by a pathologist on examination of specimens stained with NFR under a stereomicroscope, was thinly sliced with a razor knife, surgical knife or laser microdissector, and the slices were placed in RNA extraction buffer. Usually, an area >50 mm^2^ was shaved to maintain at least 80% cancer cells. Cancer cells were stained a darker red by NFR compared with the normal cells. Cancer tissue was cut out on the basis of the staining pattern, and specimens stained with HE served as a reference.

### RNA extraction and circular DNA (cDNA) synthesis

Proteinase was added to a cancer-cell suspension, and the mixture was heated to cause cytolysis. RNA was refined by simple column extraction or by phenol extraction and ethanol precipitation, and cDNA synthesis was synthesized using random hexamer as a primer.

### Analysis by quantitative reverse transcription (RT) polymerase chain reaction (PCR)

Formalin-fixed 10-μm thick paraffin-embedded sections of resected primary colorectal cancer tumors were obtained from identified areas with the highest tumor concentration and were then mounted onto uncoated glass slides. For histological diagnosis, representative sections were stained with haematoxylin and eosin using standard methods. Prior to microdissection, sections were stained with nuclear fast red (American MasterTech, Lodi, CA, USA). The sections were selectively isolated by laser capture microdissection (P.A.L.M. Microsystem; Leica, Wetzlar, Germany), according to standard procedures (13). The dissected tissues were transferred to a reaction tube containing 400 μl RNA lysis buffer (Invitrogen Life Technologies, Carlsbad, CA, USA).

The samples were homogenised at 92°C for 30 min. A total of 50 μm of 2 M sodium acetate (pH 4.0) was added, followed by 600 μl phenol/chloroform/isoamyl alcohol (250:50:1). The tubes were vortexed for 15 sec, placed on ice for 15 min, and then centrifuged at 16,000 × g for 8 min at 8°C centrifuge. The upper aqueous phase was removed and placed in a 1.5 ml centrifuge tube. A total of 10 μl glycogen and 300–400 μl isopropanol were added and the samples were vortexed for 10–15 sec. The tubes were chilled at −20°C for 30–45 min to precipitate the RNA. The samples were then washed in 500 μl 75% v/v ethanol and air-dried for 15 min. The pellet was resuspended in 50 μl 5 mM Tris buffer (Sigma-Aldrich, St. Louis, MO, USA). Finally, cDNA was prepared as described by Lord et al (14). Quantification of the 12 genes of interest and an internal reference gene (β-actin) was performed using a fluorescence-based real-time detection method (ABI PRISM 7900 Sequence Detection System; Applied Biosystems, Foster City, CA, USA). The PCR reaction mixture consisted of 120 nM of each primer, 200 nM probe, 0.4 U/l of AmpliTaq gold polymerase, 200 nM of each dATP, dCTP, dGTP, dTTP, 3.5 mM MgCl_2_ and 1× Taqman buffer, containing a reference dye (Applied Biosystems). The final volume of the reaction mixture was 20 μl. PCR conditiosn were as follows: 50°C for 2 min and 95°C for 10 min, followed by 46 cycles of 95°C for 15 sec and 60°C for 1 min. The primers and probe used were as follows: Forward, 5′-GCCTCGGTGTGCCTTTCA-3′ and reverse, 5′-CCCGTGATGTGCGCAAT-3′ for TS; and Taqman probe 5′-TCGCCAGCTACGCCCTGCTCA-3′; and forward 5′-AGGACGCAAGGAGGGTTTG-3′ and reverse, 5′-GTCCGCCGAGTCCTTACTGA-3′ for DPD; and Taqman probe 5′-CAGTGCCTACAGTCTCGAGTCTGCCAGT-3′; forward, 5′-CCTGCGGACGGAATCCT-3′ and reverse, 5′-GCTGTGATGAGTGGCAGGCT-3′for TP; and Taqman probe 5′-CAGCCAGAGATGTGACAGCCACCGT-3′; foward, 5′-GGCTGGAGGAGACCAAGGAT-3′ and reverse, 5′-CATGAGTGTCAGGAAGCGGA-3′ for FPGS; and Taqman probe 5′-CAGCTGTGTCTCCATGCCCCCCTAC-3′; forward, 5′-GCGAGCCTCGAGCTGTCTA-3′ and reverse, 5′-AATATTCCGATGATGGGCTTCTT-3′ for GGH; and Taqman probe 5′-ACCCCACGGCGACACCGC-3′; forward, 5′-GTCCTCCCGCTGCTGTCA-3′ and reverse, 5′-GCCGATGCCCATGTTCTG-3′ for DHFR; and Taqman probe 5′-TTCGCTAAACTGCATCGTCGCTGTGTC-3′.

### Calculation of results (DTP values)

Genes are amplified two-fold on every cycle of PCR. Gene expression values (relative mRNA levels) are expressed as ratios (differences between Ct values) of the gene of interest and the internal reference gene (β-actin). Therefore, the gene expression ratio of each sample is calculated as a 2-Ct value. The quantity of cancer cells removed from each specimen differs and was therefore expressed relative to the expression of β-actin expression to correct for differences in cell quantities. In addition, correction coefficients were calculated on the basis of the results of the analysis of standard samples containing known concentrations of target genes. The measured values were multiplied by the correction coefficients to derive DTP values, which were regarded as expression levels of the target genes. DTP values were calculated by the following formula, in which dCt is the Ct value of the target gene minus the Ct value of β-actin, and K is the correction coefficient: DTP = K × 2^−dCt^.

### Statistical methods

Disease-free survival (DFS), measured as the interval from the date of surgery to the date of the first documented evidence of recurrence, death or a second cancer, was calculated using the Kaplan-Meier method. The associations between the mRNA expression levels of the various metabolizing enzymes and the clinicopathological factors of age, gender, invasion depth, lymph-node metastasis, disease stage and tumor location were tested by Wilcoxon’s test. The median mRNA expression level of each metabolizing enzyme was regarded as the cutoff value, and tumors were classified as having high or low expression as compared with this value. The associations between the high and low expression levels of each enzyme and DFS were analyzed using Kaplan-Meier curves, and differences between survival curves were computed with the log-rank test. P<0.05 was considered to indicate a statistically significant difference.

## Results

### Patient characteristics

Between February 2004 and June 2006, a complete resection with no microscopically residual tumor (R0) was performed in 54 patients, who subsequently received oral S-1. The mRNA levels of the tumors were measured. Table I shows the demographic characteristics of the patients. The median age was 67 years (range, 31–84 years). The primary lesion was located in the colon or rectosigmoid colon in 40 patients (74.1%) and in the rectum in 14 (25.9%). Overall, 16 patients (29.6%) exhibited stage II disease and 38 (70.4%) exhibited stage III disease.

### Clinicopathological factors versus TS, DPD, TP, FPGS, GGH and DHFR mRNA levels

The mRNA expression levels [median (range)] of TS, DPD, FPGS, GGH and DHFR are shown in Table II. There was no correlation between the mRNA expression levels of any of these enzymes and any of the clinicopathological factors of age, gender, primary site, location, invasion depth or lymph-node metastasis (Table III).

A correlation analysis of TS, DPD and TP, three enzymes involved in 5-FU metabolism, showed a significant positive correlation between TP and DPD (data not shown; Spearman’s correlation coefficient, 0.78; P<0.0001).

### DFS versus mRNA levels of TS, DPD, TP, FPGS, GGH and DHFR

**Figure 1:**
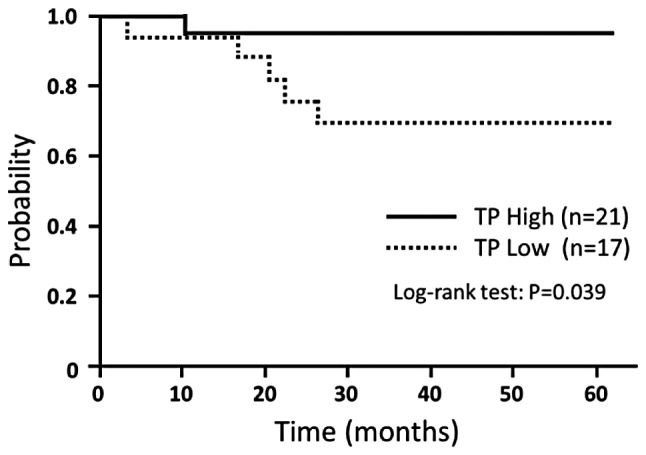
Kaplan-Meier plot of disease-free survival for stage III colorectal cancer patients according to thymidine phosphorylase (TP) expression level.

DFS did not differ significantly between the patients with high mRNA expression and those with low mRNA expression of any factor associated with the sensitivity to various types of anticancer agents in the study group as a whole, but there was a trend toward a longer DFS in the patients with high TP expression (P=0.066). According to disease stage, no factor was associated with survival in the patients with stage II disease. However, in the patients with stage III disease who received post-operative adjuvant chemotherapy, there was a statistical difference between the association of low TP expression levels and DFS compared with high TP expression levels (P=0.039) (Fig. 1). DFS did not differ significantly according to the expression level of any other factor (Table IV).

## Discussion

S-1, an oral 5-FU-based anticancer drug, is indicated for the treatment of seven types of cancer in Japan, including gastric cancer, colorectal cancer, and head and neck cancer (15). S-1 is also approved in various countries in Asia and Europe. S-1 has been found to be at least as effective as conventional 5-FU-based anticancer agents and was designed to reduce gastrointestinal toxicity, an adverse reaction specifically associated with 5-FU analogues. S-1 contains gimeracil, which strongly inhibits DPD, a metabolizing enzyme of 5-FU derivatives, thereby maintaining high concentrations of 5-FU in serum (16). In addition, S-1 contains oteracil potassium, which inhibits the phosphorylation of 5-FU in the gastrointestinal tract, an important cause of gastrointestinal toxicity, and thereby inhibits adverse effects (17). Our previous study analyzed the safety and effectiveness of a one-year treatment with S-1 in patients with resected stage II or III colorectal cancer. The treatment completion rate was 77.7%, and watery eyes was the only grade 3 or higher adverse reaction (1 patient). The three-year DFS rate was 85%, showing that S-1 is safe and effective (9). At present, the usefulness of S-1 as a post-operative adjuvant chemotherapy is being evaluated in phase III clinical trials in patients with colorectal cancer, and S-1 may become a standard treatment for colorectal cancer in the future (18).

The present study measured the mRNA expression levels of TS, DPD, TP, FPGS, GGH and DHFR, enzymes that are important in the chemotherapy of colorectal cancer with 5-FU-based agents, and examined the associations between such levels and DFS. TS, an enzyme required for DNA synthesis, is a target enzyme of 5-FU. DPD is an enzyme that affects the pharmacokinetics of 5-FU. TP is not only involved in 5-FU metabolism, but is also known as a platelet-derived endothelial cell growth factor, which has angiogenic activity (19–21). Several studies have demonstrated that tumors with low levels of TS, DPD and TP gene expression are more sensitive to 5-FU, not only in advanced or recurrent colorectal cancer, but also in gastric cancer and breast cancer (22–25). In particular, TP expression levels have been shown to differ by a factor of 2.6 times between patients who are more sensitive and those who are less sensitive to chemotherapy (22).

Few studies have examined the correlations between TP expression and the clinical usefulness of post-operative adjuvant chemotherapy in patients with colorectal cancer. Sadahiro *et al* (26) found that post-operative adjuvant chemotherapy with uracil and tegafur (UFT)/leucovorin is beneficial in patients with colorectal cancer and high TP expression levels, and reported that TP expression levels may be a useful predictor of treatment response. Another study showed that high TP expression was associated with a significantly higher survival rate in patients with Duke’s C colorectal cancer who received 5′-deoxy-5-fluorouridine (5′-DFUR) (27). Since TP is an enzyme that not only participates in 5-FU metabolism, but also converts 5′-DFUR to 5-FU, it was proposed as a potential predictor of response. By contrast, experimental studies also reported that high TP expression is associated with the decreased sensitivity of colorectal cancer to 5-FU (28,29), and certain clinical trials found no clinically useful correlation between TP expression and the response to post-operative adjuvant chemotherapy with agents such as 5-FU/leucovorin and 5′-DFUR (30,31). The ability to use TP mRNA expression to predict response to post-operative adjuvant chemotherapy in patients with colorectal cancer thus remains controversial.

In the present study, high TP expression was associated with good outcomes, particularly in the patients with stage III disease. These findings and the results of a previous study by Sadahiro *et al* (26) showing that high TP expression is associated with good outcomes in patients who received UFT/leucovorin suggest that the mechanism of action and clinical effects of post-operative adjuvant chemotherapy with S-1, containing uracil and gimeracil, which prevents 5-FU catabolism by inhibiting DPD, or with regimens that include UFT, differ from those of other 5-FU-based anticancer agents (26). As S-1 and UFT enhance serum 5-FU concentrations by inhibiting DPD, the response to these drugs may be more susceptible to catalytic reactions mediated by TP than other 5-FU analogues.

The present results demonstrated a significant positive correlation between TP and DPD expression. This finding was consistent with the result of a study by Collie-Duguid *et al* (32), which reported a positive correlation between TP and DPD expression in colorectal cancer. In the present study, however, outcomes similar to those in patients with high TP expression were not obtained in patients with high DPD expression. One of the reasons for this finding may be that S-1 was clinically effective regardless of DPD expression.

In conclusion, the present study measured the mRNA expression levels of factors associated with the sensitivity to various types of anticancer agents and found that TP is a predictor of response. The results suggest that TP can be used to predict the response to post-operative adjuvant chemotherapy with S-1. However, as the number of patients was small, firm conclusions could not be drawn. Further large clinical studies of factors associated with sensitivity to various types of anticancer agents are required to confirm these findings.

## Figures and Tables

**Table I:** Patient characteristics.

Parameter	Value
Age, years	
Median (range)	67 (31–84)
Gender, n (%)	
Male	39 (72.2)
Female	15 (27.8)
Primary site, n (%)	
Colon/rectosigmoid	40 (74.1)
Rectum	14 (25.9)
Invasion depth, n (%)	
MP	1 (1.9)
SM	1 (1.9)
SS	21 (38.9)
SE	14 (25.9)
SI	4 (7.4)
A	13 (24.1)
Lymph node metastasis, n (%)	
N0	16 (29.6)
N1	23 (42.6)
N2	12 (22.2)
N3	3 (5.6)
Stage, n (%)	
II	16 (29.6)
III	38 (70.4)

MP, muscularis propria; SM, submucosa; SS, subserosa; SE, serosal exposure; SI, serosal invasion; A, adventitia.

**Table II:** Expression level of mRNA (n=54).

Molecular markers	Median (range)
TS	4.075 (1.100–20.48)
DPD	0.295 (0.050–1.080)
TP	2.575 (0.880–23.94)
FPGS	0.590 (0.220–1.370)
GGH	12.961 (1.600–166.5)
DHFR	5.380 (1.620–12.53)

mRNA expression is relative to β-actin expression levels. TS, thymidylate synthase; DPD, dihydropyrimidine dehydrogenase; TP, thymidine phosphorylase; FPGS, folypolyglutamate synthetase; GGH, γ-glutamyl hydrolase; DHFR, dihydrofolate reductase.

**Table III:** Association between the clinicophathological factors and TS, DPD, TP, FPGS, GGH and DHFR mRNA levels.

	TS		DPD		TP		FPGS		GGH		DHFR	
						
Parameter	Median	P-value	Median	P-value	Median	P-value	Median	P-value	Median	P-value	Median	P-value
Age, years												
<65	4.33	0.664	0.27	0.631	2.15	0.632	0.56	0.112	14.7	0.228	5.25	0.811
≥65	3.91		0.30		2.84		0.62		10.6		5.69	
Gender												
Male	4.11	0.678	0.29	0.354	2.49	0.315	0.57	0.167	12.6	0.839	5.18	0.150
Female	3.95		0.32		3.58		0.65		14.4		6.39	
Primary site												
Colon/rectosigmoid	4.01	0.782	0.32	0.459	2.75	0.813	0.59	0.502	12.3	0.093	5.38	0.441
Rectum	4.24		0.24		2.46		0.60		23.1		5.35	
Invasion depth												
MP	4.08	0.990	0.40	0.846	2.93	0.651	0.76	0.631	21.9	0.417	6.27	0.885
SM	3.95		0.27		2.14		0.35		5.46		6.39	
SS	4.86		0.24		2.11		0.56		12.2		5.31	
SE	3.79		0.32		2.96		0.60		13.7		5.22	
SI	5.77		0.41		3.75		0.50		9.87		5.96	
A	4.11		0.25		2.71		0.62		23.0		4.77	
Lymph node metastasis												
Node(−)	4.91	0.293	0.20	0.172	2.20	0.198	0.58	0.563	10.2	0.229	5.06	0.820
Node(+)	3.94		0.32		2.85		0.59		15.6		5.38	

mRNA expression is relative to β-actin expression levels. TS, thymidylate synthase; DPD, dihydropyrimidine dehydrogenase; TP, thymidine phosphorylase; FPGS, folypolyglutamate synthetase; GGH, γ-glutamyl hydrolase; DHFR, dihydrofolate reductase; MP, muscularis propria; SM, submucosa; SS, subserosa; SE, serosal exposure; SI, serosal invasion; A, adventitia.

**Table IV:** Association between DFS and TS, DPD, TP, FPGS, GGH and DHFR mRNA levels.

	All patients			Stage III		
		
Molecular marker	n	3-year DFS, %	P-value	n	3-year DFS, %	P-value
TS						
High (≥4.075)	27	85.2	0.847	17	88.2	0.628
Low (<4.075)	27	85.2		21	81.0	
DPD						
High (≥0.295)	27	92.6	0.310	21	90.1	0.272
Low (<0.295)	27	77.3		17	75.3	
TP						
High (≥2.575)	27	96.3	0.066	21	95.2	0.039
Low (<2.575)	27	73.6		17	69.3	
FPGS						
High (≥0.590)	27	88.9	0.313	20	90.0	0.283
Low (<0.590)	27	81.2		18	77.0	
GGH						
High (≥12.96)	27	88.7	0.311	21	85.5	0.803
Low (<12.96)	27	81.5		17	82.4	
DHFR						
High (≥5.380)	27	81.5	0.636	19	80.0	0.338
Low (<5.380)	27	88.6		19	88.9	

mRNA expression is relative to β-actin expression levels. DFS, disease-free survival; TS, thymidylate synthase; DPD, dihydropyrimidine dehydrogenase; TP, thymidine phosphorylase; FPGS, folypolyglutamate synthetase; GGH, γ-glutamyl hydrolase; DHFR, dihydrofolate reductase.
